# The genome sequence of *Veronica serpyllifolia* L., 1753 (Lamiales: Plantaginaceae)

**DOI:** 10.12688/wellcomeopenres.26891.1

**Published:** 2026-06-27

**Authors:** Markus Ruhsam, Royal Botanic Garden Edinburgh Genome Acquisition Lab, Plant Genome Sizing Collective, Darwin Tree of Life Barcoding collective, Wellcome Sanger Institute Tree of Life Management, Samples and Laboratory team, Wellcome Sanger Institute Scientific Operations: Sequencing Operations, Wellcome Sanger Institute Tree of Life Core Informatics team, Tree of Life Core Informatics collective, Darwin Tree of Life Consortium

**Affiliations:** 1 Royal Botanic Garden Edinburgh, Edinburgh, Scotland, UK

**Keywords:** Veronica serpyllifolia, Thyme-leaved Speedwell, genome sequence, chromosomal, Lamiales

## Abstract

We present a genome assembly of *Veronica serpyllifolia* (Thyme-leaved Speedwell; Streptophyta; Magnoliopsida; Lamiales; Plantaginaceae). The assembly consists of two haplotypes with total lengths of 368.14 megabases and 347.49 megabases. Most of haplotype 1 (99.76%) is scaffolded into 7 chromosomal pseudomolecules. Haplotype 2 was assembled to scaffold level. The mitochondrial sequence has a length of 334.11 kilobases and the plastid genome assembly has a length of 150.93 kilobases. Gene annotation of this assembly on Ensembl identified 24 804 protein-coding genes. This assembly was generated as part of the Darwin Tree of Life project, which produces reference genomes for eukaryotic species found in Britain and Ireland.

## Species taxonomy

Eukaryota; Viridiplantae; Streptophyta; Streptophytina; Embryophyta; Tracheophyta; Euphyllophyta; Spermatophyta; Magnoliopsida; Mesangiospermae; eudicotyledons; Gunneridae; Pentapetalae; asterids; lamiids; Lamiales; Plantaginaceae; Veroniceae; *Veronica*; Veronica subgen. Beccabunga; *Veronica serpyllifolia* L., 1753 (NCBI:txid164258).

## Background

Thyme-leaved Speedwell, *Veronica serpyllifolia* L., is a small creeping perennial in the family Plantaginaceae. It has prostrate or decumbent stems that root at the nodes, with short ascending branches and small, opposite, broadly ovate leaves. The pale blue, bright blue or whitish flowers are borne in leafy terminal racemes and may be produced over a long period, with the main flowering season from March to October ( Botanical Society of Britain and Ireland, 2026).

The species occurs in a wide range of open, damp and often disturbed habitats, including poorer grassland, grazed meadows, fens, heaths, damp lawns, woodland clearings, track edges and waste ground. It tolerates variation in soil chemistry, moisture, shade, grazing and trampling, but is less able to persist where tall or dense vegetation closes the sward. *Veronica serpyllifolia* is native, common and widespread in Britain and Ireland, and has a circumpolar boreo-temperate distribution. It is also widely naturalised in both hemispheres ( Botanical Society of Britain and Ireland, 2026).

**Figure 1.**
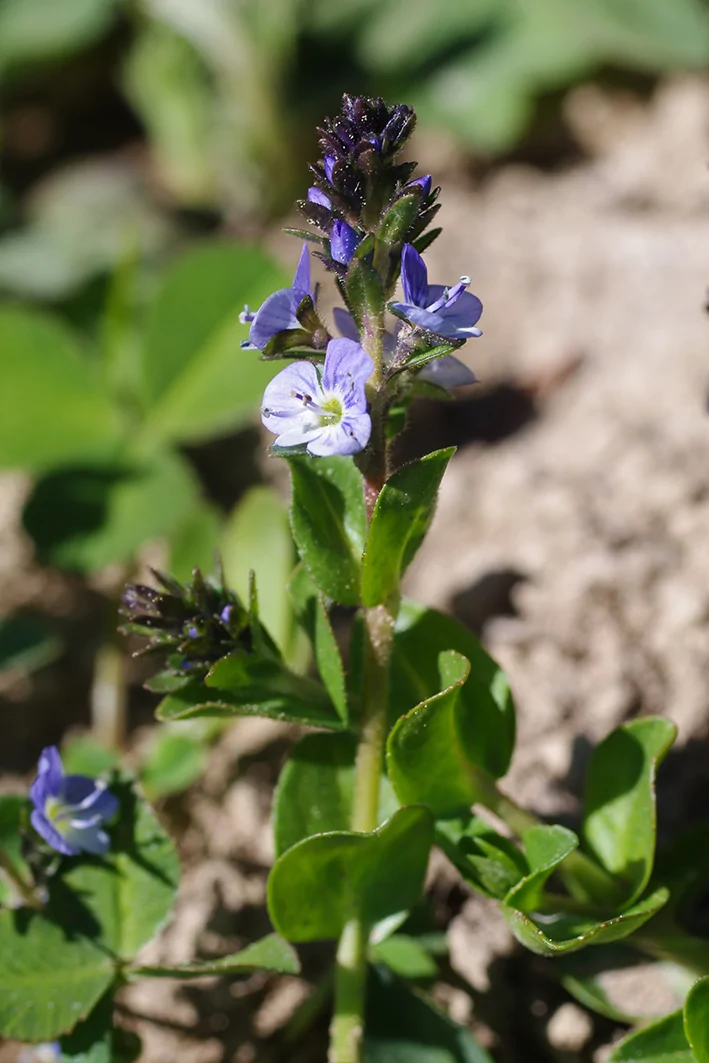
Photograph of *Veronica serpyllifolia* by Joëlle Magnin-Gonze .

We present a chromosome-level genome sequence for *Veronica serpyllifolia*, as part of the Darwin Tree of Life Project. This genome was assembled using the Tree of Life pipeline from a specimen collected in Gorebridge, United Kingdom ( Figure 1).

## Methods

### Sample acquisition, flow cytometry and DNA barcoding

A specimen of *Veronica serpyllifolia* (specimen ID EDTOL01448, ToLID daVerSerp1) was used for genome sequencing. It was collected from Gorebridge, United Kingdom (latitude 55.8432, longitude −3.0694) on 2021-06-14. Another specimen, collected on the same occasion, was used for RNA sequencing (specimen ID EDTOL01449, ToLID daVerSerp2). The specimens were collected and identified by Markus Ruhsam.

The genome size was estimated by flow cytometry following the ‘one-step’ method outlined in Pellicer *et al*. (2021) and using propidium iodide as the fluorochrome. CyStain PI OxProtect Staining Buffer (Sysmex UK Ltd) was used for isolation of nuclei ( Loureiro *et al.*, 2007), and the internal calibration standard was *Solanum lycopersicum* L. ‘Stupické polní rané’ with an assumed 1C-value of 968 Mb ( Doležel *et al.*, 2007).

The initial identification was verified by an additional DNA barcoding process according to the framework developed by Twyford *et al*. (2024). Part of the plant specimen was preserved in silica gel desiccant ( Chase & Hills, 1991). DNA extracted from the dried plant was amplified by PCR for standard barcode markers, with the amplicons sequenced and compared to public sequence databases including GenBank and the Barcode of Life Database (BOLD) ( Ratnasingham & Hebert, 2007). Following whole genome sequence generation, the relevant DNA barcode region was also used alongside the initial barcoding data for sample tracking at the WSI ( Twyford *et al.*, 2024). The standard operating procedures for Darwin Tree of Life barcoding are available on protocols.io.

### Nucleic acid extraction

Protocols for high molecular weight (HMW) DNA extraction developed at the Wellcome Sanger Institute (WSI) Tree of Life Core Laboratory are available on protocols.io ( Howard *et al.*, 2025). The daVerSerp1 sample was weighed and triaged to determine the appropriate extraction protocol. For HMW DNA extraction, 52.40 mg of tissue was used. The tissue was homogenised by cryogenic disruption using the Covaris cryoPREP® Automated Dry Pulverizer. HMW DNA was extracted using the Automated Plant MagAttract v2 protocol. Sheared DNA was purified by manual SPRI (solid-phase reversible immobilisation), using (Pacific Biosciences) AMPure PB beads to eliminate shorter fragments and concentrate the DNA.

The concentration of the sheared and purified DNA was assessed using a Nanodrop spectrophotometer and Qubit Fluorometer using the Qubit dsDNA High Sensitivity Assay kit. Fragment size distribution was evaluated by running the sample on the FemtoPulse system. For this sample, the final post-shearing DNA had a Qubit concentration of 10.5 ng/μL and a yield of 476.70 ng, with a fragment size of 15.4 kb. The 260/280 spectrophotometric ratio was 1.93, and the 260/230 ratio was 0.95. The Genomic Quality Number (GQN) was 7.7.

RNA was extracted from whole plant tissue of daVerSerp2 in the Tree of Life Laboratory at the WSI using the RNA Extraction: Automated MagMax™ *mir*Vana protocol. The RNA concentration was assessed using a Nanodrop spectrophotometer and a Qubit Fluorometer using the Qubit RNA Broad-Range Assay kit. Analysis of the integrity of the RNA was done using the Agilent RNA 6000 Pico Kit and Eukaryotic Total RNA assay.

### PacBio HiFi library preparation and sequencing

Library preparation and sequencing were performed at the WSI Scientific Operations core. Libraries were prepared using the SMRTbell Prep Kit 3.0 (Pacific Biosciences) according to the manufacturer’s instructions. The kit includes reagents for end repair/A-tailing, adapter ligation, post-ligation SMRTbell bead clean-up, and nuclease treatment. Size selection and clean-up were performed using diluted AMPure PB beads (Pacific Biosciences). DNA concentration was quantified using a Qubit Fluorometer v4.0 (ThermoFisher Scientific) and the Qubit 1X dsDNA HS assay kit. Final library fragment size was assessed with the Agilent Femto Pulse Automated Pulsed Field CE Instrument (Agilent Technologies) using the gDNA 55 kb BAC analysis kit.

The sample was sequenced using the Sequel IIe system (Pacific Biosciences, California, USA). The concentration of the library loaded onto the Sequel IIe was in the range 40–135 pM. SMRT Link software, a PacBio web-based workflow manager, was used to set up and monitor the run, and to perform primary and secondary analysis of the data upon completion.

### Hi-C

***Sample preparation and crosslinking***

Hi-C data were generated from the whole plant tissue of daVerSerp1 using the Arima-HiC v2 kit (Arima Genomics). Tissue was finely ground using the Covaris cryoPREP Dry Pulverizer (Covaris), and then subjected to nuclei isolation. Nuclei were isolated using a modified protocol based on the Qiagen QProteome Cell Compartment Kit (Qiagen), in which only the Lysis and CE2 buffers were used, with QIAshredder spin columns. After isolation, nuclei were fixed using formaldehyde to a final concentration of 2% to crosslink the DNA. The crosslinked DNA was then digested and biotinylated according to the manufacturer’s instructions. A clean-up step was performed with SPRIselect beads before library preparation. DNA concentration was quantified using the Qubit Fluorometer v4.0 (Thermo Fisher Scientific) and the Qubit HS Assay Kit, following the manufacturer’s instructions.

***Hi-C library preparation and sequencing***

Biotinylated DNA constructs were fragmented using a Covaris E220 sonicator and size selected to 400–600 bp using SPRISelect beads. DNA was enriched with Arima-HiC v2 kit Enrichment beads. End repair, A-tailing, and adapter ligation were carried out with the NEBNext Ultra II DNA Library Prep Kit (New England Biolabs), following a modified protocol where library preparation occurs while DNA remains bound to the Enrichment beads. Library amplification was performed using KAPA HiFi HotStart mix and a custom Unique Dual Index (UDI) barcode set (Integrated DNA Technologies). Depending on sample concentration and biotinylation percentage determined at the crosslinking stage, libraries were amplified with 10–16 PCR cycles. Post-PCR clean-up was performed with SPRISelect beads. Libraries were quantified using the AccuClear Ultra High Sensitivity dsDNA Standards Assay Kit (Biotium) and a FLUOstar Omega plate reader (BMG Labtech).

Prior to sequencing, libraries were normalised to 10 ng/μL. Normalised libraries were quantified again to create equimolar and/or weighted 2.8 nM pools. Pool concentrations were checked using the Agilent 4200 TapeStation (Agilent) with High Sensitivity D500 reagents before sequencing. Sequencing was performed using paired-end 150 bp reads on the Illumina NovaSeq 6000.

### RNA library preparation and sequencing

Libraries were prepared using the NEBNext® Ultra II Directional RNA Library Prep Kit for Illumina (New England Biolabs), following the manufacturer’s instructions. Poly(A) mRNA was isolated from 100 ng of total RNA using oligo (dT) beads, converted to cDNA, and uniquely indexed; 14 PCR cycles were performed. Libraries were prepared with an intended fragment size of 100–300 bp. Libraries were quantified, normalised, and pooled equimolarly to a final concentration of 2.8 nM before dilution to 150 pM for loading. Sequencing was carried out on the Illumina NovaSeq 6000, generating paired-end reads.

### Genome assembly

Prior to assembly of the PacBio HiFi reads, a database of *k*-mer counts ( *k* = 31) was generated from the filtered reads using FastK. GenomeScope2 ( Ranallo-Benavidez *et al.*, 2020) was used to analyse the *k*-mer frequency distributions, providing estimates of genome size, heterozygosity, and repeat content.

The HiFi reads were assembled using Hifiasm in Hi-C phasing mode ( Cheng *et al.*, 2021, 2022), producing two haplotypes. Hi-C reads ( Rao *et al.*, 2014) were mapped to the primary contigs using bwa-mem2 ( Vasimuddin *et al.*, 2019). Contigs were further scaffolded with Hi-C data in YaHS ( Zhou *et al.*, 2023), using the --break option for handling potential misassemblies. The scaffolded assemblies were evaluated using Gfastats ( Formenti *et al.*, 2022), BUSCO ( Manni *et al.*, 2021) and MerquryFK ( Rhie *et al.*, 2020). The organelle genomes were assembled using OATK ( Zhou *et al.*, 2025).

### Assembly curation

The assembly was decontaminated using the Assembly Screen for Cobionts and Contaminants ( ASCC) pipeline. TreeVal was used to generate the flat files and maps for use in curation. Manual curation was conducted primarily in PretextView and HiGlass ( Kerpedjiev *et al.*, 2018). Scaffolds were visually inspected and corrected as described by Howe *et al*. (2021). Manual corrections included six breaks and 117 joins. This reduced the scaffold count by 76.5% and increased the scaffold N50 by 1.8%. The curation process is described at https://gitlab.com/wtsi-grit/rapid-curation . PretextSnapshot was used to generate a Hi-C contact map of the final assembly.

### Assembly quality assessment

The MerquryFK tool ( Rhie *et al.*, 2020) was run in a Singularity container ( Kurtzer *et al.*, 2017) to evaluate *k*-mer completeness and assembly quality for both haplotypes using the *k*-mer database ( *k* = 31) computed prior to genome assembly. The analysis outputs included assembly QV scores and completeness statistics.

The genome was analysed using the BlobToolKit pipeline, a Nextflow implementation of the earlier Snakemake version ( Challis *et al.*, 2020). The pipeline aligns PacBio reads using minimap2 ( Li, 2018) and SAMtools ( Danecek *et al.*, 2021) to generate coverage tracks. It runs BUSCO ( Manni *et al.*, 2021) using lineages identified by querying NCBI datasets ( O’Leary *et al.*, 2024). For the three domain-level lineages, BUSCO genes are aligned to the UniProt Reference Proteomes database ( Bateman *et al.*, 2023) using DIAMOND blastp ( Buchfink *et al.*, 2021). The genome is divided into chunks based on the density of BUSCO genes from the closest taxonomic lineage, and each chunk is aligned to the UniProt Reference Proteomes database with DIAMOND blastx. Sequences without hits are chunked using seqtk and aligned to the NT database with blastn ( Altschul *et al.*, 1990). The BlobToolKit suite consolidates all outputs into a blobdir for visualisation. The BlobToolKit pipeline was developed using nf-core tooling ( Ewels *et al.*, 2020) and MultiQC ( Ewels *et al.*, 2016), with containerisation through Docker ( Merkel, 2014) and Singularity ( Kurtzer *et al.*, 2017).

## Genome sequence report

### Sequence data

**Figure 2.**
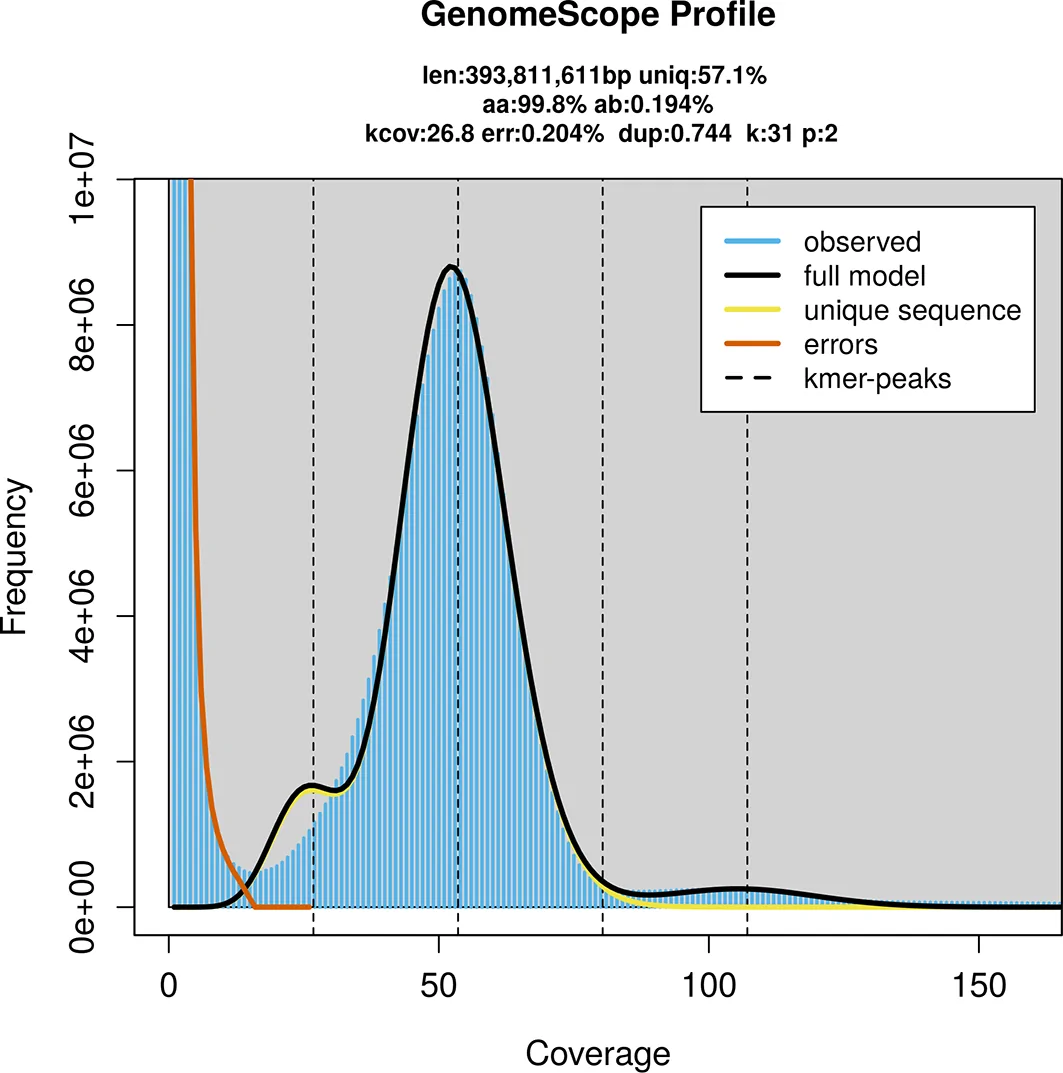
Frequency distribution of *k*-mers generated using GenomeScope2. The plot shows observed and modelled *k*-mer spectra, providing estimates of genome size, heterozygosity, and repeat content based on unassembled sequencing reads.

The genome of a specimen of *Veronica serpyllifolia* was sequenced using Pacific Biosciences single-molecule HiFi long reads, generating 22.52 Gb (gigabases) from 1.62 million reads, which were used to assemble the genome. GenomeScope2.0 analysis estimated the haploid genome size at 394.26 Mb, with a heterozygosity of 0.19% and repeat content of 42.89% ( Figure 2). Using flow cytometry, the genome size (1C-value) of the sample was estimated to be 0.45 pg, equivalent to 440.00 Mb. These estimates guided expectations for the assembly. Based on the estimated genome size, the sequencing data provided approximately 54× coverage. Hi-C sequencing produced 83.89 Gb from 277.79 million reads, which were used to scaffold the assembly. RNA sequencing data were also generated and are available in public sequence repositories. Table 1 summarises the specimen and sequencing details.

**Table 1.** Specimen and sequencing data for *Veronica serpyllifolia* (BioProject PRJEB76343).

Platform	PacBio HiFi	Hi-C	RNA-seq
**ToLID**	daVerSerp1	daVerSerp1	daVerSerp2
**Specimen ID**	EDTOL01448	EDTOL01448	EDTOL01449
**BioSample (source individual)**	SAMEA10983545	SAMEA10983545	SAMEA10983546
**BioSample (tissue)**	SAMEA10983654	SAMEA10983654	SAMEA10983655
**Tissue**	whole plant	whole plant	whole plant
**Instrument**	Sequel IIe	Illumina NovaSeq 6000	Illumina NovaSeq 6000
**Run accessions**	ERR13245269; ERR13245268	ERR13248931	ERR13248932
**Read count total**	1.62 million reads	277.79 million read pairs	34.82 million read pairs
**Base count total**	22.52 Gb	83.89 Gb	10.52 Gb

### Assembly statistics

The genome was assembled into two haplotypes using Hi-C phasing. Haplotype 1 was curated to chromosome level, while haplotype 2 was assembled to scaffold level. The final assembly has a total length of 368.14 Mb in 30 scaffolds, with 174 gaps, and a scaffold N50 of 58.54 Mb ( Table 2).

**Table 2.** Genome assembly statistics for *Veronica serpyllifolia.*

Genome assembly	Haplotype 1	Haplotype 2
**Assembly name**	daVerSerp1.hap1.1	daVerSerp1.hap2.1
**Assembly accession**	GCA_964197965.1	GCA_964197985.1
**Assembly level**	chromosome	scaffold
**Span (Mb)**	368.14	347.49
**Number of chromosomes**	7	-
**Number of contigs**	204	129
**Contig N50**	6.49 Mb	6.24 Mb
**Number of scaffolds**	30	54
**Scaffold N50**	58.54 Mb	51.26 Mb
**Longest scaffold length (Mb)**	67.3	64.68
**Organelles**	Mitochondrial genome: 334.11 kb; Plastid genome: 150.93 kb	-

**Figure 3.**
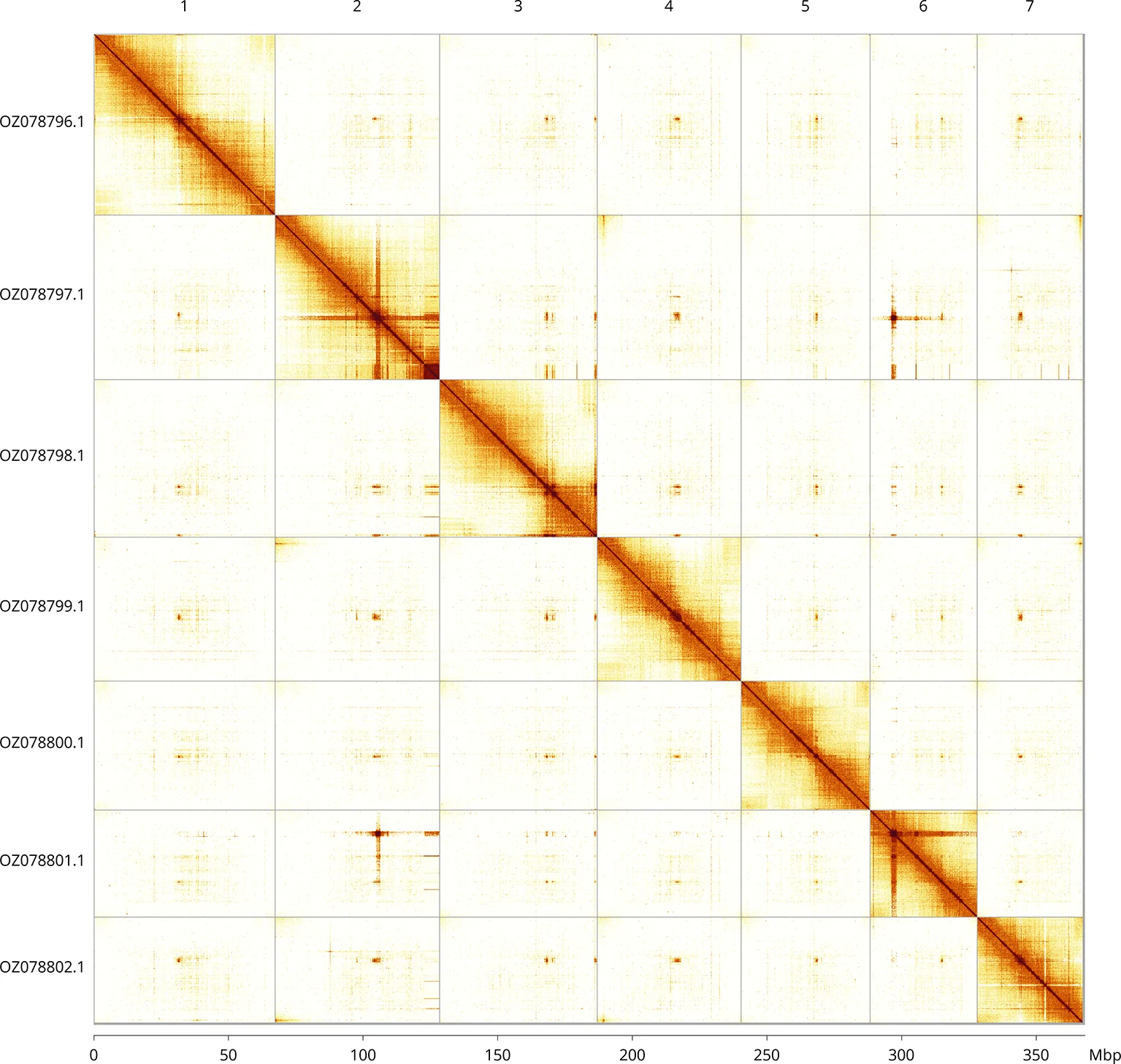
Hi-C contact map of the *Veronica serpyllifolia* genome assembly. Assembled chromosomes are shown in order of size and labelled along the axes, with a megabase scale shown below. The plot was generated using PretextSnapshot.

Most of the assembly sequence (99.76%) was assigned to 7 chromosomal-level scaffolds. These chromosome-level scaffolds, confirmed by Hi-C data, are named according to size ( Figure 3; Table 3). The region on Chromosome 2 from ~55 Mbp to the end is of uncertain order and orientation.

**Table 3.** Chromosomal pseudomolecules in the haplotype 1 genome assembly of *Veronica serpyllifolia* daVerSerp1.

INSDC accession	Molecule	Length (Mb)	GC%
OZ078796.1	1	67.30	36.50
OZ078797.1	2	61.07	38.50
OZ078798.1	3	58.54	36.50
OZ078799.1	4	53.43	35.50
OZ078800.1	5	47.84	35.50
OZ078801.1	6	39.85	36.50
OZ078802.1	7	39.22	36

The mitochondrial genome (length 334.11 kb, OZ078803.1) and plastid genome (length 150.93 kb, OZ078804.1) were also assembled. These sequences are included as contigs in the multifasta file of the genome submission and as standalone records.

### Assembly quality metrics

**Figure 4.**
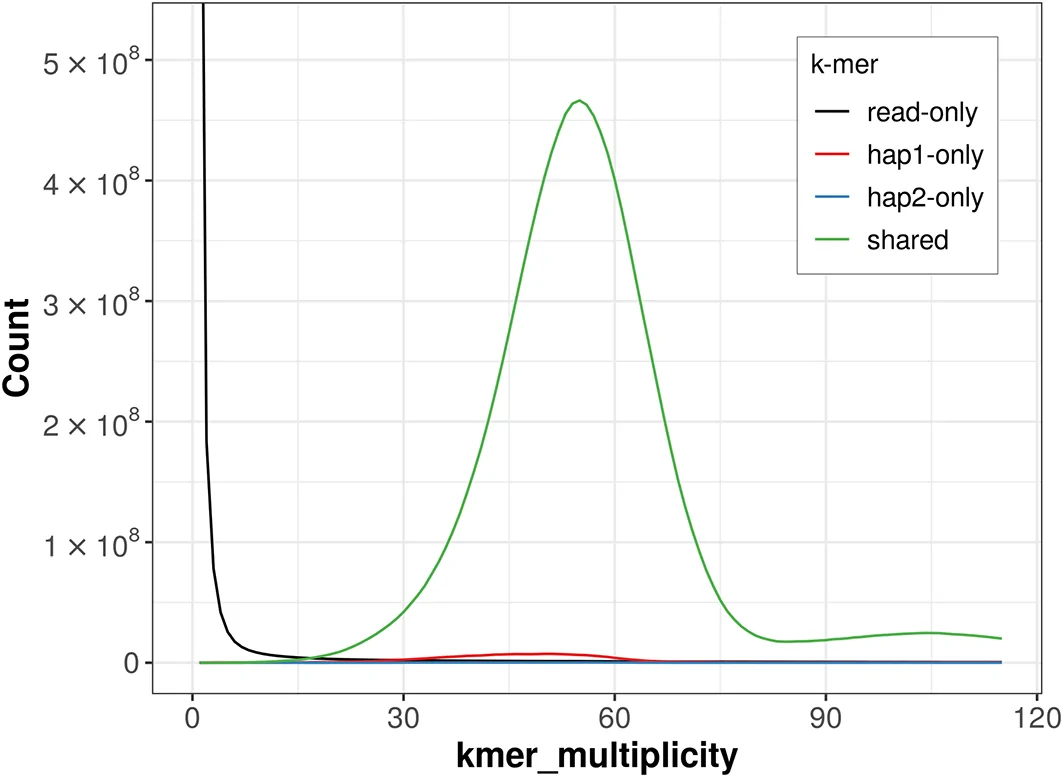
Evaluation of *k*-mer completeness using MerquryFK. This plot illustrates the recovery of *k*-mers from the original read data in the final assemblies. The horizontal axis represents *k*-mer multiplicity, and the vertical axis shows the number of *k*-mers. The black curve represents *k*-mers that appear in the reads but are not assembled. The green curve corresponds to *k*-mers shared by both haplotypes, and the red and blue curves show *k*-mers found only in one of the haplotypes.

For haplotype 1, the estimated QV is 65.7, and for haplotype 2, 66.9. When the two haplotypes are combined, the assembly achieves an estimated QV of 66.2. The *k*-mer completeness is 98.42% for haplotype 1, 96.44% for haplotype 2, and 98.49% for the combined haplotypes ( Figure 4).

**Figure 5.**
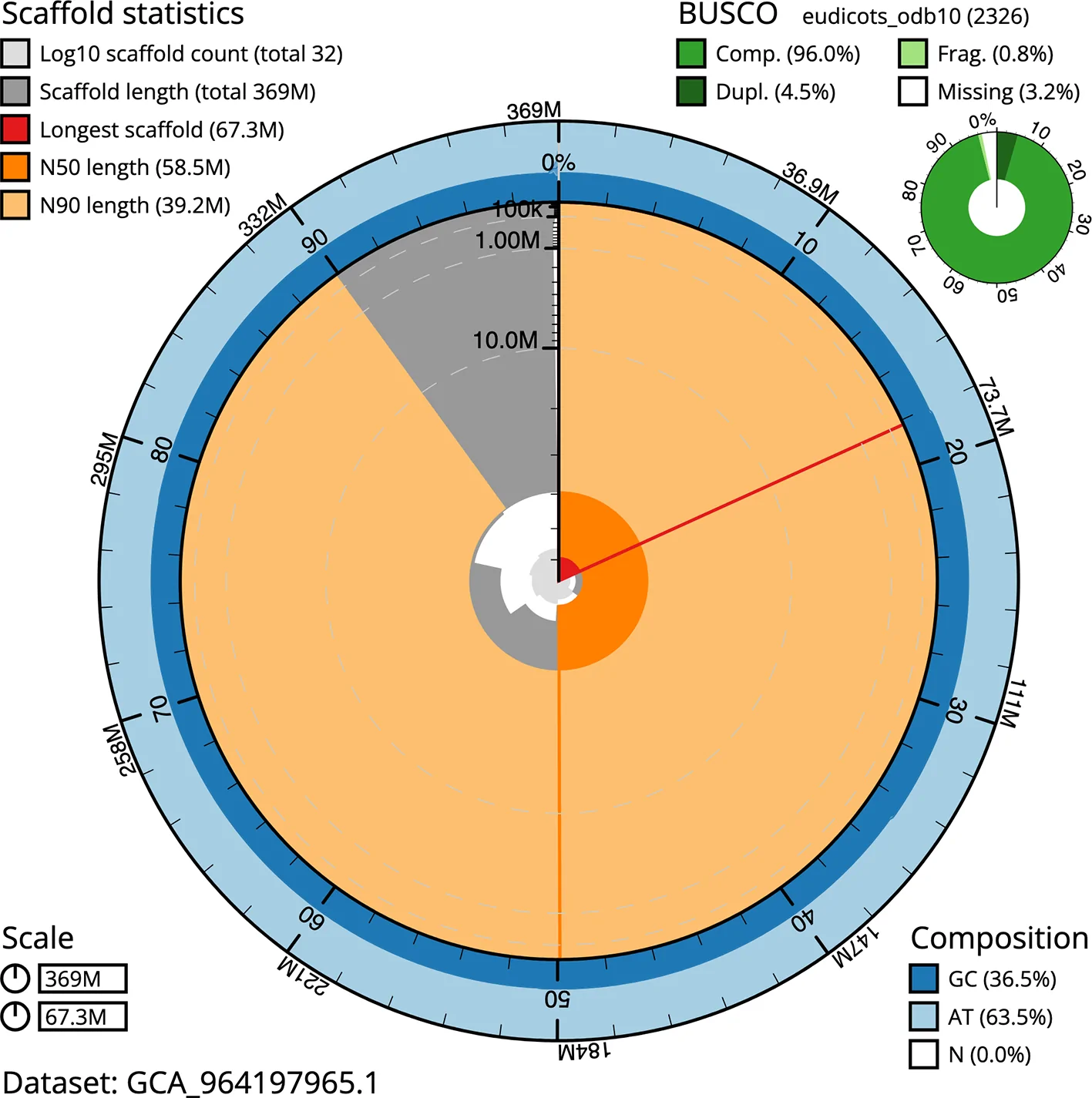
Assembly metrics for daVerSerp1.hap1.1. The BlobToolKit snail plot provides an overview of assembly metrics and BUSCO gene completeness. The circumference represents the length of the whole genome sequence, and the main plot is divided into 1 000 bins around the circumference. The outermost blue tracks display the distribution of GC, AT, and N percentages across the bins. Scaffolds are arranged clockwise from longest to shortest and are depicted in dark grey. The longest scaffold is indicated by the red arc, and the deeper orange and pale orange arcs represent the N50 and N90 lengths. A light grey spiral at the centre shows the cumulative scaffold count on a logarithmic scale. A summary of complete, fragmented, duplicated, and missing BUSCO genes in the set is presented at the top right. An interactive version of this figure can be accessed on the BlobToolKit viewer.

**Figure 6.**
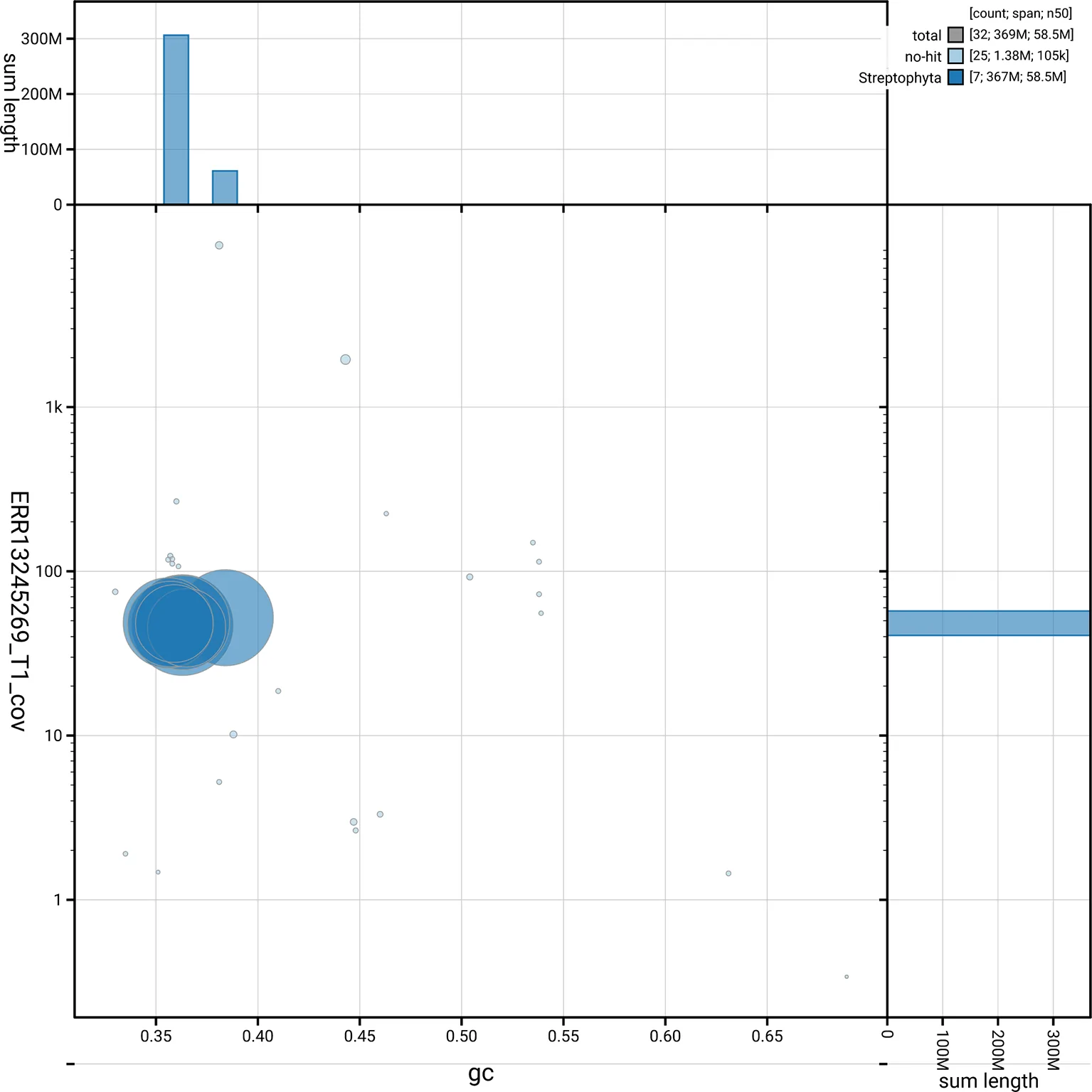
BlobToolKit blob plot for daVerSerp1.hap1.1. The plot shows base coverage (vertical axis) and GC content (horizontal axis). The circles represent scaffolds, with the size proportional to scaffold length and the colour representing phylum membership. The histograms along the axes display the total length of sequences distributed across different levels of coverage and GC content. An interactive version of this figure is available on the BlobToolKit viewer.

BUSCO analysis using the eudicots_odb10 reference set ( *n* = 2 326) identified 96.0% of the expected gene set (single = 91.5%, duplicated = 4.5%) for haplotype 1. The snail plot in Figure 5 summarises the scaffold length distribution and other assembly statistics for haplotype 1. The blob plot in Figure 6 shows the distribution of scaffolds by GC proportion and coverage for haplotype 1.

Table 4 lists the assembly metric benchmarks adapted from Rhie *et al.* (2021) and the Earth BioGenome Project Report on Assembly Standards January 2026. The EBP metric calculated for the haplotype 1 is **6.C.Q65**, meeting the recommended 6.C.Q40 reference standard.

**Table 4.** Earth biogenome project summary metrics for the *Veronica serpyllifolia* assembly.

Measure	Value	Benchmark
EBP summary (haplotype 1)	6.C.Q65	6.C.Q40
Contig N50 length	6.49 Mb	≥ 1 Mb
Scaffold N50 length	58.54 Mb	= chromosome N50
Consensus quality (QV)	Haplotype 1: 65.7; haplotype 2: 66.9; combined: 66.2	≥ 40
*k*-mer completeness	Haplotype 1: 98.42%; Haplotype 2: 96.44%; combined: 98.49%	≥ 95%
BUSCO	C:96.0% [S:91.5%, D:4.5%], F:0.8%, M:3.2%, n:2 326	S > 90%; D < 5%
Percentage of assembly assigned to chromosomes	99.76%	≥ 90%

**Notes:** The EBP summary uses log10(Contig N50); chromosome-level (C) or log10(Scaffold N50); Q (Merqury QV). BUSCO: C = complete; S = single-copy; D = duplicated; F = fragmented; M = missing; n = orthologues.

**Table 5.** Software versions and sources used for *Veronica serpyllifolia.*

Software	Version	Source
BLAST	2.14.0	ftp://ftp.ncbi.nlm.nih.gov/blast/executables/blast+/
BlobToolKit	4.3.9	https://github.com/blobtoolkit/blobtoolkit
BUSCO	5.5.0	https://gitlab.com/ezlab/busco
bwa-mem2	2.2.1	https://github.com/bwa-mem2/bwa-mem2
DIAMOND	2.1.8	https://github.com/bbuchfink/diamond
fasta_windows	0.2.4	https://github.com/tolkit/fasta_windows
FastK	1.1	https://github.com/thegenemyers/FASTK
GenomeScope2.0	2.0.1	https://github.com/tbenavi1/genomescope2.0
Gfastats	1.3.6	https://github.com/vgl-hub/gfastats
Hifiasm	0.19.8-r603	https://github.com/chhylp123/hifiasm
HiGlass	1.13.4	https://github.com/higlass/higlass
MerquryFK	1.1.0-c1	https://github.com/thegenemyers/MERQURY.FK
Minimap2	2.24-r1122	https://github.com/lh3/minimap2
Oatk	0.9	https://github.com/c-zhou/oatk
MultiQC	1.14; 1.17 and 1.18	https://github.com/MultiQC/MultiQC
Nextflow	23.10.0; 23.10.1	https://github.com/nextflow-io/nextflow
PretextSnapshot	0.0.4	https://github.com/sanger-tol/PretextSnapshot
PretextView	1.0.3	https://github.com/sanger-tol/PretextView
samtools	1.16.1; 1.17; 1.18; 1.19.2; 1.6	https://github.com/samtools/samtools
sanger-tol/ascc	0.1.0	https://github.com/sanger-tol/ascc
sanger-tol/blobtoolkit	0.6.0	https://github.com/sanger-tol/blobtoolkit
sanger-tol/curationpretext	1.4.2	https://github.com/sanger-tol/curationpretext
Seqtk	1.4-r122	https://github.com/lh3/seqtk
Singularity	3.9.0	https://github.com/sylabs/singularity
TreeVal	1.1.0	https://github.com/sanger-tol/treeval
YaHS	1.2a.2	https://github.com/c-zhou/yahs

## Genome annotation report

The *Veronica serpyllifolia* genome assembly (GCA_964197965.1) was annotated by Ensembl at the European Bioinformatics Institute (EBI). This annotation includes 61 295 transcribed mRNAs from 24 804 protein-coding and 26 265 non-coding genes. The average transcript length is 1 930.08 bp, with an average of 1.20 coding transcripts per gene and 3.86 exons per transcript. For further information about the annotation, please refer to the annotation page on Ensembl.

## Author information

• Members of the Royal Botanic Garden Edinburgh Genome Acquisition Lab
• Members of the Plant Genome Sizing Collective
• Members of the Darwin Tree of Life Barcoding collective
• Members of the Wellcome Sanger Institute Tree of Life Management, Samples and Laboratory team
• Members of Wellcome Sanger Institute Scientific Operations – Sequencing Operations
• Members of the Wellcome Sanger Institute Tree of Life Core Informatics team
• Members of the Tree of Life Core Informatics collective
• Members of the Darwin Tree of Life Consortium

## Wellcome Sanger Institute – Legal and Governance

The materials that have contributed to this genome note have been supplied by a Darwin Tree of Life Partner. The submission of materials by a Darwin Tree of Life Partner is subject to the **‘Darwin Tree of Life Project Sampling Code of Practice’**, which can be found in full on the Darwin Tree of Life website. By agreeing with and signing up to the Sampling Code of Practice, the Darwin Tree of Life Partner agrees they will meet the legal and ethical requirements and standards set out within this document in respect of all samples acquired for, and supplied to, the Darwin Tree of Life Project. Further, the Wellcome Sanger Institute employs a process whereby due diligence is carried out proportionate to the nature of the materials themselves, and the circumstances under which they have been/are to be collected and provided for use. The purpose of this is to address and mitigate any potential legal and/or ethical implications of receipt and use of the materials as part of the research project, and to ensure that in doing so we align with best practice wherever possible. The overarching areas of consideration are:

• Ethical review of provenance and sourcing of the material
• Legality of collection, transfer and use (national and international)

Each transfer of samples is further undertaken according to a Research Collaboration Agreement or Material Transfer Agreement entered into by the Darwin Tree of Life Partner, Genome Research Limited (operating as the Wellcome Sanger Institute), and in some circumstances, other Darwin Tree of Life collaborators.

## Data Availability

European Nucleotide Archive: Veronica serpyllifolia. Accession number PRJEB76343; https://identifiers.org/ena.embl/PRJEB76343. The genome sequence is released openly for reuse. The *Veronica serpyllifolia* genome sequencing initiative is part of the Darwin Tree of Life Project (PRJEB40665), the Sanger Institute Tree of Life Programme (PRJEB43745) and AEGIS (PRJEB80366). All raw sequence data and the assembly have been deposited in INSDC databases. Raw data and assembly accession identifiers are reported in Tables 1 and 2. Pipelines used for genome assembly at the WSI Tree of Life are available at https://pipelines.tol.sanger.ac.uk/pipelines. Table 5 lists software versions used in this study.
