# Supplementary material for: The genome sequence of Veronica serpyllifolia L., 1753 (Lamiales: Plantaginaceae)
Source: Wellcome Open Res. 2026 Jun 27;11:403. [Version 1] doi: 10.12688/wellcomeopenres.26891.1 (PMC13598394; doi:10.12688/wellcomeopenres.26891.1)
Supplement: Peer review history

# Reviewer report: Reviewer response for version 1

**DOI:** 10.21956/wellcomeopenres.29610.r168854

**Published:** 2026-10-07

**Authors:** Itzi Fragoso-Martínez

**Affiliations:** 1 Red de Biodiversidad y Sistemática, Instituto de Ecología A.C., Xalapa,, Veracruz, Mexico

The authors present the genome sequence of *Veronica serpyllifolia* (Plantaginaceae), along with its chromosome number and organelle sequences. The manuscript is concise and well-written. However, I have two minor suggestions and one inquiry for the authors to address:

1. **Voucher Information:** In the Methods section, the authors state that a specimen of *V. serpyllifolia* was collected, but no voucher information is provided (e.g., collector name, collection number, or herbarium acronym). If tracking voucher specimens is not a strict requirement of the Tree of Life Project, this comment may be disregarded.
2. **Structural Variants/Uncertain Regions:** Please expand the information regarding the 55 Mbp region of chromosome 2 whose orientation remains uncertain.
3. **Comparative Context:** Have other species from the Plantaginaceae family or the broader Lamiales order been sequenced as part of this project? If so, incorporating a brief comparison of chromosome numbers or organelle genome sizes would provide valuable context for the findings.

Are sufficient details of methods and materials provided to allow replication by others?

Yes

Is the rationale for creating the dataset(s) clearly described?

Yes

Are the datasets clearly presented in a useable and accessible format?

Yes

Are the protocols appropriate and is the work technically sound?

Yes

Reviewer Expertise:

phylogenomics, phylogenetics, botany, plant taxonomy

I confirm that I have read this submission and believe that I have an appropriate level of expertise to confirm that it is of an acceptable scientific standard.

## References

[1] Singh AP. Peer Review Report For: The genome sequence of Veronica serpyllifolia L., 1753 (Lamiales: Plantaginaceae) [version 1; peer review: 2 approved]. Wellcome Open Res 2026, 11:403 (https://doi.org/10.21956/wellcomeopenres.29610.r159911).10.12688/wellcomeopenres.26891.1PMC1359839442787000

# Reviewer report: Reviewer response for version 1

**DOI:** 10.21956/wellcomeopenres.29610.r168861

**Published:** 2026-09-22

**Authors:** Samuel J. Smit

**Affiliations:** 1 University of York, York, England, UK

The dataset presented is for *Veronica serpyllifolia*, a species within the Lamiales. The author describes the species sequenced but the rationale as to why this species was sequenced is not clear. Although native to Britain and Ireland, it is widely distributed across the world and often considered to be a weed. More information on, for example, its ecological significance and/or use as a medicinal herb will be a welcome addition to the introduction.

 The methods and assembly are clearly described except for a minor omission under the nucleic acid extraction section where a few suppliers and/or manufacturers for the reagents, kits and instruments used are not specified. 

 Assembly data is accessible and complete.

Are sufficient details of methods and materials provided to allow replication by others?

Partly

Is the rationale for creating the dataset(s) clearly described?

Partly

Are the datasets clearly presented in a useable and accessible format?

Yes

Are the protocols appropriate and is the work technically sound?

Yes

Reviewer Expertise:

plant genome assembly, comparative genomics

I confirm that I have read this submission and believe that I have an appropriate level of expertise to confirm that it is of an acceptable scientific standard.

# Reviewer report: Reviewer response for version 1

**DOI:** 10.21956/wellcomeopenres.29610.r159911

**Published:** 2026-07-31

**Authors:** Aditya Pratap Singh

**Affiliations:** 1 Bidhan ChandraKrishi Viswavidyalaya, Kalyani, India; 2 Assistant Breeder, Comienzo Agri Science Limited, Kharora, Chhattisgarh, India

Rationale & Background-

 Strengths: The background gives a solid botanical description, habitat preferences, and geographic distribution for V. serpyllifolia.

 Technical Soundness & Methodology-

 Strengths: The technical pipeline represents the state-of-the-art for plant genome assembly. The use of flow cytometry to benchmark genome size prior to assembly is excellent practice.

 Minor Technical Clarifications-

 Genome Size Gap: There is a noticeable difference between the flow cytometry estimate. A brief comment explaining this ~70 Mb difference (e.g., whether it is likely due to unresolved pericentromeric heterochromatin, rDNA arrays, or unplaced contigs in Haplotype 2) would benefit assembly specialists.

 The author states that "The region on Chromosome 2 from ~55 Mbp to the end is of uncertain order and orientation." It would be helpful to briefly state what caused this ambiguity (e.g., low Hi-C read coverage, complex repeat structure, or potential structural heterozygosity).

 Reproducibility & Data Accessibility-

 Sample identifiers (ToLID daVerSerp1/daVerSerp2, BioSample SAMEA114389025), precise GPS coordinates, tissue weights, and DNA/RNA extraction metrics (Qubit, FemtoPulse, GQN) are thoroughly documented.

 Standard Operating Procedures (SOPs) are linked via protocols.io, and software versions are comprehensively detailed in Table 5. The dataset is fully accessible across INSDC (BioProject PRJEB76343), Ensembl, and the BlobToolKit viewer.

 Actionable Recommendations for Author-

 Kindly add 1–2 sentences in the Genome sequence report section discussing the difference between the flow cytometry estimate and the assembled haploid genome. Kindly clarify the cause of the orientation/order uncertainty at the terminus of Chromosome 2 (~55 Mbp onwards).

 The author may note that the low estimated heterozygosity likely contributed to the high phasing efficiency and smooth assembly into 7 clear Hi-C linkage groups.

Are sufficient details of methods and materials provided to allow replication by others?

Yes

Is the rationale for creating the dataset(s) clearly described?

Yes

Are the datasets clearly presented in a useable and accessible format?

Yes

Are the protocols appropriate and is the work technically sound?

Yes

Reviewer Expertise:

Genetics, Crop improvement, corn breeding, MAS, multi-omics

I confirm that I have read this submission and believe that I have an appropriate level of expertise to confirm that it is of an acceptable scientific standard.
